# Association study between the polymorphisms of angiogenesis‐related genes and cervical cancer susceptibility in Chinese Uygur population

**DOI:** 10.1002/mgg3.899

**Published:** 2019-09-02

**Authors:** Lili Han, Sulaiya Husaiyin, Chunhua Ma, Mayinuer Niyazi

**Affiliations:** ^1^ Department of Gynecology People’s Hospital of Xinjiang Uygur Autonomous Region Urumqi China

**Keywords:** cervical cancer, genetics polymorphisms, *VEGF‐C*, *VEGFR‐2*, *VEGFR‐3*

## Abstract

**Background:**

Cervical cancer is the second most common malignant tumor in women, and its invasion and metastasis are regulated by tumor angiogenic growth factors and their cognate receptors. In this study, we explored the relationship between genetic polymorphisms of angiogenesis‐related genes (*VEGF‐C*, *VEGFR‐2*, and *VEGFR‐3*) and the risk of cervical cancer in Chinese Uygur population.

**Methods:**

We investigated four single‐nucleotide polymorphisms (SNPs) in 342 cervical cancer cases and 498 controls to evaluate their association with the risk of cervical cancer. Their correlations were evaluated by chi‐squared test, Fisher's exact test, *t* test, and genetic model analyses. Odds ratios (ORs) and 95% confidence intervals (95% CIs) were calculated using unconditional logistic regression.

**Results:**

We observed that rs12646659 in *VEGF‐C* was associated with a lower cervical cancer risk in allele, dominant, and log‐additive models (allele: *p* = .017; dominant: *p* = .018; log‐additive: *p* = .018). For the individuals older than 43, rs4604006 (*VEGF‐C*) was related to an increased cervical cancer risk under codominant model (*p* = .035), and rs12646659 was significantly associated with a reduced cervical cancer risk in allele, dominant, log‐additive models (allele: *p* = .028; codominant: *p* = .037; log‐additive: *p* = .037) However, there were no significant correlation of rs1000611 (*VEGFR‐2*) and rs1195571 (*VEGFR‐3*) with cervical cancer risk in Chinese Uygur population.

**Conclusion:**

Our study firstly provided evidence that rs4604006 and rs12646659 of *VEGF‐C* gene were related to the susceptibility of cervical cancer in Chinese Uygur population.

## 1 INTRODUCTION

Cervical cancer is one of the common malignant tumor in women, with 528,000 cases and 266,000 deaths in 2012 (Fang et al., 2017; Niu et al., 2019). The high incidence and mortality of cervical cancer among Uygur women in Xinjiang has become the most important health issue (Lin, Huang, Shen, & Yiming, 2015; Ma, Hong, Lu, Chen, & Ma, 2015). In recent years, the incidence of cervical cancer among women in Xinjiang is on the rise. The chances of the second cure for cervical cancer are very low, and the early detection has a significant impact on the survival of cervical cancer patients (Waggoner, 2003). Increasing growth factors and their homologous receptors have been reported and can regulate the invasion and metastasis of cervical cancer (Tomao et al., 2014). Therefore, the research of these growth factors and their receptors is undoubtedly a great benefit for the treatment of cervical cancer.

Angiogenesis is a pivotal step in tumor formation, growth and metastasis (Wu, 2014). *VEGF* (vascular endothelial growth factor) is a key angiogenic stimulator, and VEGF signaling pathway has been identified as an important part of angiogenesis (Li, Xu, Gao, Bi, & Huo, 2018). *VEGF‐C* (Vascular Endothelial Growth Factor C; OMIM: 601,528) is an important member of the *VEGF* family. It encodes proteins that affect angiogenesis, endothelial cell growth, and vascular permeability (Chen et al., 2014). Recent studies have shown that elevated levels of vascular *VEGF‐C* have in many invasive tumors and it is strongly associated with poor prognosis in cancer patients (Cheng, Jiang, Yuan, Liu, & Simoncini, 2018). For example, the level of *VEGF‐C* increases in women with lung carcinoma, and it is significantly associated with lymph node metastasis (Tamura & Ohta, 2003). The high expression of *VEGF‐C* was also observed in cervical cancer (Mitsuhashi et al., 2005). At the same time, VEGF binds to the extracellular receptor domain and promotes the activation of tyrosine kinase in the intracellular receptor domain, thereby phosphorylating tyrosine residues and activating several intracellular signaling pathways (Robinson & Stringer, 2001).

There are three types of VEGF receptors: *VEGFR‐1* (vascular endothelial growth factor receptor 1), *VEGFR‐2* (vascular endothelial growth factor receptor 2; OMIM: 191,306), and *VEGFR‐3* (vascular endothelial growth factor receptor 3; OMIM: 136,352). *VEGFR‐2* receptors are predominantly expressed in vascular endothelial cells (Shibuya, 2006), while *VEGFR‐3* is especially expressed in endothelial lymphatic cells (Hamrah et al., 2004). The *VEGFR‐3* gene, also known as *FLT4* (fms‐related tyrosine kinase 4), has a molecular weight of 195 kDa (Takahashi & Shibuya, 2005). *Flt‐4* has been found to be expressed in a variety of human malignancies (Su et al., 2006). *VEGFR‐2* also known as KDR (kinase insert domain receptor), has a molecular weight of 230 kDa (Takahashi & Shibuya, 2005). Studies have shown that *VEGF‐C* and *VEGF‐D* bind to its receptor *VEGFR‐2* (*KDR*) and receptor *VEGFR‐3* (*Flt‐4*), promoting angiogenesis and/or lymphangiogenesis, thus accelerates tumor growth and metastasis (Joukov et al., 1996). These evidences suggested that *VEGF‐C*, *VEGFR‐2,* and *VEGFR‐3* were closely related to the growth and metastasis of cervical cancer.

In this case–control study, we genotyped four SNPs (rs10006115 [*VEGFR‐2*], rs4604006 [*VEGF‐C*], rs12646659 [*VEGF‐C*], and rs11955717 [*VEGFR‐3*]) and performed a comprehensive association analysis to identify whether SNPs were associated with cervical cancer risk in Chinese Uygur population.

## 2 MATERIALS AND METHODS

### 2.1 Ethics statement

The study was approved by the ethics committee of People's Hospital of Xinjiang Uygur Autonomous Region, in accordance with the principles of the Helsinki Declaration. Each participant was informed of the procedures and purpose of our research and signed a written informed consent before donating 5 ml venous blood for further analyses.

### 2.2 Research participates

In this case–control study, 342 cervical cancer patients (mean age, 43.27 ± 11.78 years) were recruited from the Department of Gynecology, People's Hospital of Xinjiang Uygur Autonomous Region, between 2016 and 2019. All patients were diagnosed with cervical cancer by histopathological examination according to the International Federation of Gynecology and Obstetrics (FIGO; Du, Wang, Richards, & Wang, 2019).

The healthy control group consisted of 498 individuals (43.46 ± 13.03 years), who were recruited from the Health Examination Center of the People's Hospital of Xinjiang Uygur Autonomous Region during the same time with cases. And these control subjects were comparable to the cervical cancer subjects in terms of age and race. The selection criteria for the control group were as follows: no history of cancer or a family history of cancer, no known history of infectious HPV. Written informed consent was obtained from each participant prior to enrollment in the study.

### 2.3 SNP selection and genotyping

Genomic DNA was extracted from participant's peripheral venous blood by a Gold Mag Mini Whole Blood Genomic DNA Purification Kit (GoldMag Ltd) following the manufacturer's protocol and then stored at −80°C before genotyping. The concentration and purity of DNAs were determined by the NanoDrop 2000 (Thermo Scientific).

We established the following criteria to identify the target SNPs: (a) MAF (minor allele frequency) of Han Chinese in Beijing (HCB) > 0.05 and disease relevance in 1,000 genome (http://www.internationalgenome.org/); (b) a linkage disequilibrium value of *r* ^2^ < .8 for each target SNPs. Agena MassARRAY Assay Design 4.0 software was used to design the primers for amplification and extension reactions. Agena MassARRAY RS1000 was used to perform SNP genotyping according to the standard protocol. Two staffs independently operated genotyping assay and randomly selected more than 10% samples for verification, and the results were exactly same in two sets of assays. Then, Agena Typer 4.0 software was applied to analyze and manage our data. The PCR primers for each SNP are shown in Table 1.

**Table 1:** Primers used for this study

SNP_ID	2nd PCRP	1st PCRP	UEP DIR	UEP SEQ
rs10006115	ACGTTGGATGATGGCAGCCATATGGAGTTG	ACGTTGGATGTCCACTGCAGAAGAAATGGC	R	aggaTCTTCTCTCTGCCAACTC
rs4604006	ACGTTGGATGACATACTCATGTTTGTACCC	ACGTTGGATGGTGTGGCATGAAACAAAAGC	R	ggagGTTTGTACCCAATCCTTTG
rs11955717	ACGTTGGATGCGTCACCAAACAGCATTTCG	ACGTTGGATGGAGGTTTGAATTTACGTGGC	R	CAAGGTAATTAAGTAAAAGGTGTC
rs12646659	ACGTTGGATGAAGCTTGGCTGTCTCTATGG	ACGTTGGATGATGTTCCCACTGAGAACAAC	R	GTCTCTATGGTTATATCTTCAAATA

Abbreviations: PCR, polymerase chain reaction; UEP, unextended mini‐sequencing primer.

### 2.4 Statistical analysis

All statistical analysis was done with SPSS version 19.0 software (SPSS) and Microsoft Excel. All analyses were two sided, and statistical significance was set at *p* ＜ .05. SNP genotype frequencies in the case and controls were calculated by chi‐squared test (Hu, Wang, Hu, & Li, 2018; Yang et al., 2018). Deviation from Hardy–Weinberg equilibrium (HWE) was assessed using the chi‐squared test to compare the observed and expected genotype frequencies among the control subjects. Logistic regression analysis was used to examine the odds ratios (ORs) and 95% confidence intervals (95% CIs) in order to assess the association between SNPs and cervical cancer (Bland & Altman, 2000; Liu et al., 2017). Four models (codominant, dominant, recessive, and log‐additive) were used to test the association between SNPs and Cervical cancer (Jin et al., 2016; Sole, Guino, Valls, & Iniesta, 2006).

## 3 RESULTS

### 3.1 Demographic characteristics

The general characteristics were listed in Table 2. Among the 840 participants, 342 were patients with cervical cancer and 498 were healthy controls. The mean age and standard deviation were 43.27 ± 11.78 for cases and 43.46 ± 13.03 for control subjects. There were no significant differences between the cases and controls in terms of age.

**Table 2:** General characteristics the of this study population

Variables	Case	Control	Total	*p‐*value
Total	342	498		
Age				
≤43	166 (49%)	235 (47%)	401	>.05
>43	176 (51%)	263 (53%)	439	
Mean age ± *SD*	43.27 ± 11.78	43.46 ± 13.03		
T stage				
III + IV	80 (23%)			
I + II	132 (39%)			
Unavailable	130 (38%)			

Note

*p‐*values were calculated from two‐sided chi‐squared test/Fisher's exact test; *p* < .05 indicates statistical significance.

### 3.2 Hardy–Weinberg equilibrium and SNPs alleles

Basic information containing SNP ID, alleles, role, MAF distribution, *p*‐HWE value, ORs, 95% CIs of all candidate SNPs were presented in Table 3. The call rate for all SNPs was above 95% in cases and controls, which was considered as high quality to perform association analyses. None of the candidate SNPs significantly deviated from HWE. OR = 1 indicates that the factor had no effect on the disease; OR > 1 means it is a risk factor; and OR < 1 means it is a protective factor. The comparison of allele distributions between the cervical cancer patients and the control subjects with the χ^2^‐test revealed that there was a statistical correlation between the rs12646659 polymorphism of *VEGF‐C* and the risk reduction of cervical cancer (OR = 0.41, 95% CI = 0.20–0.87; *p* = .017).

**Table 3:** Basic information about candidate SNPs and association with the risk of cervical cancer in allele model

SNP	Chr	Position	Gene(s)	Role	Alleles	Frequency (MAF)		*p* ‐ HWE	OR (95% CI)	*p‐*value
						Cases	Controls			
rs10006115	4	55,080,187	*VEGFR−2*	ncRNA_intronic	G/T	0.056	0.049	.107	1.14 (0.74–1.76)	.563
rs4604006	4	176,687,621	*VEGF‐C*	ncRNA_intronic	C/T	0.342	0.365	.067	0.90 (0.74–1.11)	.326
rs12646659	4	176,764,117	*VEGF‐C*	Intronic	C/G	0.013	0.031	1.000	0.41 (0.20–0.87)	**.017** *
rs11955717	5	180,606,639	*VEGFR−3*	Intronic	C/T	0.329	0.336	.070	0.97 (0.79–1.19)	.752

Abbreviations: 95% CI, 95% confidence interval; Alleles A/B, Minor/Major alleles; HWE, Hardy–Weinberg equilibrium; MAF, minor allele frequency; OR, odds ratio; SNP, single‐nucleotide polymorphism.

*p*‐values were calculated with Pearson's χ^2^ tests.

* *p* < .05 indicates statistical significance.

### 3.3 Associations between genotype frequencies and cervical cancer risk

Furthermore, we analyzed the association between the SNPs and the risk of cervical cancer under multiple inheritance models (codominant, dominant, recessive, log‐additive models; Table 4). Our analyses showed that rs12646659 in *VEGF‐C* gene was correlated with a decrease the risk of cervical cancer in the dominant model (OR = 0.40, 95% CI = 0.19–0.86, *p* = .018 for the “C/G‐G/G” genotype) and log‐additive model (OR = 0.40, 95% CI = 0.19–0.86, *p* = .018) before and after adjustment for age, respectively. In addition, we failed to find any significant association between other polymorphisms and the risk of cervical cancer.

**Table 4:** Logistic regression analysis of the association between prominent SNPs and cervical cancer risk

Gene	SNP	Model	Genotype	Control	Case	Without adjustment		With adjustment	
						OR (95% CI)	*p*‐value	OR (95% CI)	*p*‐value
*VEGFR−2*	rs10006115	Codominant	G/G	452 (90.8%)	305 (89.2%)	1.00		1.00	
			G/T	43 (8.6%)	36 (10.5%)	1.24 (0.78–1.98)	.364	1.24 (0.78–1.98)	.363
			T/T	3 (0.6%)	1 (0.3%)	0.49 (0.05–4.77)	.542	0.49 (0.05–4.78)	.543
		Dominant	G/G	452 (90.8%)	305 (89.2%)	1.00	.451	1.00	.449
			G/T‐T/T	46 (9.2%0	37 (10.8)	1.19 (0.76–1.88)		1.19 (0.76–1.88)	
		Recessive	G/G‐G/T	495 (99.4%)	341 (99.7%)	1.00	.530	1.00	.531
			T/T	3 (0.6%)	1 (0.3%)	0.48 (0.05–4.67)		0.48 (0.05–4.68)	
		Log‐additive	—	—	—	1.13 (0.74–1.73)	.572	1.13 (0.74–1.73)	.570
*VEGF‐C*	rs4604006	Codominant	C/C	210 (42.2%)	146 (42.7%)	1.00		1.00	
			C/T	212 (42.6%)	158 (46.2%)	1.07 (0.80–1.44)	.644	1.07 (0.80–1.44)	.640
			T/T	76 (15.3%)	38 (11.1%)	0.72 (0.46–1.12)	.145	0.72 (0.46–1.12)	.143
		Dominant	C/C	210 (42.2%)	146 (42.7%)	1.00	.881	1.00	.882
			T/C‐C/C	288 (57.8%)	196 (57.3%)	0.98 (0.74–1.29)		0.98 (0.74–1.29)	
		Recessive	C/C ‐T/C	422 (84.7%)	304 (88.9%)	1.00	.086	1.00	.084
			T/T	76 (15.3%)	38 (11.1%)	0.69 (0.46–1.05)		0.69 (0.46–1.05)	
		Log‐additive	—	—	—	0.91 (0.74–1.11)	.336	0.91 (0.74–1.11)	.335
	rs12646659	Codominant	C/C	464 (93.5%)	333 (97.4%)	1.00		1.00	
			G/C	31 (6.3%)	9 (2.6%)	—		—	
			G/G	0 (0.0%)	0 (0.0%)	—		—	
		Dominant	C/C	464 (93.5%)	333 (97.4%)	1.00	**.019** *	1.00	**.018** *
			C/G‐G/G	31 (6.3%)	9 (2.6%)	0.40 (0.19–0.86)		0.40 (0.19–0.86)	
		Recessive	C/C‐G/C	495 (100.0%)	342 (100.0%)	1.00		1.00	
			G/G	0 (0.0%)	0 (0.0%)	—		—	
		Log‐additive	—	—	—	0.40 (0.19–0.86)	**.019** *	0.40 (0.19–0.86)	**.018** *
*VEGFR−3*	rs11955717	Codominant	T/T	227 (45.9%)	157 (45.9%)	1.00		1.00	
			T/C	203 (41.0%)	145 (42.4%)	1.03 (0.77–1.39)	.830	1.03 (0.77–1.39)	.828
			C/C	65 (13.1%)	40 (11.7%)	0.89 (0.57–1.39)	.606	0.89 (0.57–1.39)	.609
		Dominant	T/T	227 (45.9%)	157 (45.9%)	1.00	.989	1.00	.992
			T/C‐C/C	268 (54.1%)	185 (54.1%)	1.00 (0.76–1.32)		1.00 (0.76–1.32)	
		Recessive	T/T‐T/C	430 (86.9%)	302 (88.3%)	1.00	.538	1.00	.541
			C/C	65 (13.1%)	40 (11.7%)	0.88 (0.58–1.33)		0.88 (0.58–1.34)	
		Log‐additive	—	—	—	0.97 (0.79–1.18)	.759	0.97 (0.79–1.19)	.763

Abbreviations: 95% CI, 95% confidence interval; Alleles A/B, Minor/Major alleles; HWE, Hardy–Weinberg equilibrium; MAF, minor allele frequency; OR, odds ratio; SNP, single‐nucleotide polymorphism.

*p*‐values were calculated with Pearson's χ^2^ tests.

* *p* < .05 indicates statistical significance.

### 3.4 Stratification analysis by age

To further explore the potential effect of age on the relationship of *VEGF‐C*, *VEGFR‐2,* and *VEGFR‐3* gene polymorphisms with the susceptibility to cervical cancer, we performed the same statistical analysis on the recruited population after stratification of age (Table 5). Among the individuals older than 43, rs4604006 of *VEGF‐C* gene was correlated with a 1.55‐fold increased the risk of cervical cancer in the codominant model (adjusted, OR = 1.55, 95% CI = 1.03–2.34, *p* = .035 for the “C/T” genotype). While rs1264665 of *VEGF‐C* was related to a decreased the risk of cervical cancer under the allele model (OR = 0.27, 95% CI, 0.08–0.94; *p* = .028 for the “C” allele) and dominant model (adjusted, OR = 0.26, 95% CI = 0.08–0.92, *p* = .037 for the “C/G‐G/G” genotype). The variant rs1264665 was also observed to decrease the risk of cervical cancer in the log‐additive model (adjusted, OR = 0.26, 95% CI = 0.08–0.92, *p* = .037). However, no significant association between candidate polymorphisms and cervical cancer risk was found in populations at age ≤43.

**Table 5:** Relationship of prominent SNPs with the cervical cancer risk stratified by age

Gene	SNP	Model	Genotype	Age ≤ 43				Age > 43			
				Control	Case	OR (95% CI)	*p*‐value	Control	Case	OR (95% CI)	*p*‐value
*VEGFR−2*	rs10006115	Codominant	G/G	215 (91.5%)	149 (89.8%)	1.00		237 (90.1%)	156 (88.6%)	1.00	
			G/T	19 (8.1%)	17 (10.2%)	1.28 (0.64–2.54)	.466	24 (9.1%)	19 (10.8%)	1.19 (0.63–2.25)	.586
			T/T	1 (0.4%)	0 (0.0%)	/	.999	2 (0.8%)	1 (0.6%)	0.73 (0.07–8.11)	.795
		Dominant	G/G	215 (91.5%)	149 (89.8%)	1.00	.556	237 (90.1%)	156 (88.6%)	1.00	.644
			G/T‐T/T	20 (8.5%)	17 (10.2%)	1.21 (0.61–2.4)		26 (9.9%)	20 (11.4%)	1.16 (0.62–2.15)	
		Recessive	G/G‐G/T	234 (99.6%)	166 (100.0%)	1.00	.999	261 (99.2%)	175 (99.4%)	1.00	.784
			T/T	1 (0.4%)	0 (0.0%)	/		2 (0.8%)	1 (0.6%)	0.71 (0.06–7.95)	
		Log‐additive	—	—	—	1.14 (0.59–2.2)	.670	—	—	1.11 (0.63–1.95)	.723
		Allele	G	449 (95.5%)	315 (94.9%)	1.00	.668	498 (94.7%)	331 (94.0%)	1.00	.684
			T	21 (4.5%)	17 (5.1%)	1.15 (0.6–2.22)		28 (5.3%)	21 (6.0%)	1.13 (0.63–2.02)	
*VEGF‐C*	rs4604006	Codominant	C/C	90 (38.3%)	78 (47.0%)	1.00		120 (45.6%)	68 (38.6%)	1.00	
			C/T	108 (46.0%)	67 (40.4%)	0.7 (0.46–1.09)	.128	104 (39.6%)	91 (51.7%)	1.55 (1.03–2.34)	**.035** *
			T/T	37 (15.7%)	21 (12.6%)	0.66 (0.36–1.22)	.178	39 (14.8%)	17 (9.7%)	0.77 (0.4–1.46)	.425
		Dominant	C/C	90 (38.3%)	78 (47.0%)	1.00	.083	120 (45.6%)	68 (38.6%)	1.00	.141
			T/C‐C/C	145 (61.7%)	88 (53.0%)	0.69 (0.46–1.04)		143 (54.4%)	108 (61.4%)	1.34 (0.91–1.98)	
		Recessive	C/C ‐T/C	198 (84.3%)	145 (87.4%)	1.00	.386	224 (85.2%)	159 (90.3%)	1.00	.112
			T/T	37 (15.7%)	21 (12.6%)	0.78 (0.44–1.4)		39 (14.8%)	17 (9.7%)	0.61 (0.33–1.12)	
		Log‐additive	—	—	—	0.78 (0.59–1.04)	.097	—	—	1.04 (0.79–1.38)	.775
		Allele	T	288 (61.3%)	223 (67.2%)		.087	344 (65.4%)	227 (64.5%)		.782
			C	182 (38.7%)	109 (32.8%)	0.77 (0.58–1.04))		182 (34.6%)	125 (35.5%)	1.04 (0.78–1.38)	
	rs12646659	Codominant	C/C	218 (93.6%)	160 (96.4%)	1.00		246 (93.9%)	173 (98.3%)	1.00	
			G/C	15 (6.4%)	6 (3.6%)	/		16 (6.1%)	3 (1.7%)	/	
			G/G	0 (0.0%)	0 (0.0%)	/		0 (0.0%)	0 (0.0%)	/	
		Dominant	C/C	218 (93.6%)	160 (96.4%)	1.00	.219	246 (93.9%)	173 (98.3%)	1.00	**.037** *
			C/G‐G/G	15 (6.4%)	6 (3.6%)	0.56 (0.21–1.48)		16 (6.1%)	3 (1.7%)	0.26 (0.08–0.92)	
		Recessive	C/C‐G/C	233 (100.0%)	166 (100.0%)	1.00		262 (100.0%)	176 (100.0%)	1.00	
			G/G	0 (0.0%)	0 (0.0%)	—		0 (0.0%)	0 (0.0%)	/	
		Log‐additive	—	—	—	0.56 (0.21–1.48)	.219	—	—	0.26 (0.08–0.92)	**.037** *
		Allele	T	451 (96.8%)	326 (98.2%)	1.00	.220	508 (97.0%)	349 (99.2%)	1.00	**.028** *
			C	15 (3.2%)	6 (1.8%)	0.55 (0.21–1.44)		16 (3.0%)	3 (0.8%)	0.27 (0.08–0.94)	
*VEGFR−3*	rs11955717	Codominant	T/T	106 (45.3%)	82 (49.4%)	1.00		121 (46.4%)	75 (42.6%)	1.00	
			T/C	98 (41.9%)	68 (41.0%)	0.91 (0.59–1.38)	.614	105 (40.2%)	77 (43.8%)	1.19 (0.79–1.8)	.399
			C/C	30 (12.8%)	16 (9.6%)	0.68 (0.35–1.34)	.278	35 (13.4%)	24 (13.6%)	1.11 (0.61–2.01)	.734
		Dominant	T/T	106 (45.3%)	82 (49.4%)	1.00	.419	121 (46.4%)	75 (42.6%)	1.00	.419
			T/C‐C/C	128 (54.7%)	84 (50.6%)	0.85 (0.57–1.27)		140 (53.6%)	101 (57.4%)	1.17 (0.8–1.73)	
		Recessive	T/T‐T/C	204 (87.2%)	150 (90.4%)	1.00	.327	226 (86.6%)	152 (46.4%)	1.00	.953
			C/C	30 (12.8%)	16 (9.6%)	0.72 (0.38–1.36)		35 (13.4%)	24 (86.4%)	1.02 (0.58–1.78))	
		Log‐additiv e	—	—	—	0.85 (0.63–1.15)	.290	—	—	1.09 (0.83–1.43)	.545
		Allele	T	310 (66.2%)	232 (69.9%)	1.00	.278	347 (66.5%)	227 (64.5%)	1.00	.544
			C	158 (33.8%)	100 (30.1%)	0.85 (0.62–1.15)		175 (33.5%)	125 (35.5%)	1.09 (0.82–1.45)	

Abbreviations: 95% CI; 95% confidence interval; Alleles A/B, Minor/Major alleles; HWE, Hardy–Weinberg equilibrium; MAF, minor allele frequency; OR, odds ratio; SNP, single‐nucleotide polymorphism.

*p*‐values were calculated by unconditional logistic regression analysis with adjustments for age.

“/”means no data.

* *p* < .05 indicates statistical significance.

## 4 DISCUSSION

In this hospital‐based case–control study, we investigated the association of four important polymorphisms (rs10006115, rs4604006, rs12646659, and rs11955717) with the risk of cervical cancer in Chinese Uygur population, and we observed a significant association between the *VEGF‐C* rs12646659 polymorphism and the risk of cervical cancer. The presence of the *VEGF‐C* rs12646659 conferred a lower risk of developing cervical cancer. Further stratified analysis revealed that rs4604006 of *VEGF‐C* gene was related to a higher risk of cervical cancer at age >43, while rs12646659 of *VEGF‐C* was associated with a lower risk of cervical cancer at age >43. Our present study is the first to provide substantial basic evidence that gene polymorphisms in *VEGF‐C* were corrected with cervical cancer risk in Chinese Uygur population.

Due to tumor growth and metastasis require the formation of new blood vessels, blocking tumor angiogenesis can be used to treat cancer. Meanwhile, genes related to angiogenesis also become potential target molecules for tumor treatment (Hajari Taheri et al., 2019). *VEGF* family proteins are specific and potent angiogenic factors that increase vessel permeability, endothelial cell growth, proliferation, migration, and differentiation (Keck et al., 1989). *VEGF‐C*, a member of the *VEGF* family, is a protein‐coding gene (Olofsson et al., 1996). *VEGF‐C* associated with Diseases, including Lymphedema, Hereditary, and Lymphedema. Recently, several reports have confirmed the correlation between *VEGF‐C* expression in tumor tissue specimens and lymph node metastasis. In cervical cancer, *VEGF‐C* mRNA expression in tumor tissue samples has been shown to be associated with lymph node metastasis (Niki et al., 2000). *VEGF‐C* tissue status also was an important independent factor for poor prognosis, and serum *VEGF* and *VEGF‐C* levels can also be used as biomarkers for cervical SCC (Mitsuhashi et al., 2005). In addition, *VEGF‐C* can binds to receptor Flt‐4 and promotes angiogenesis and/or lymphangiogenesis, thus accelerating cancer metastasis (Joukov et al., 1996; Lohela, Bry, Tammela, & Alitalo, 2009). *VEGF‐C* accelerated cervical cancer invasiveness via regulation of galectin‐3 or moesin protein expression (Liu, Cheng, He, & Yao, 2014). *VEGF‐C* can also reduce the expression of miR‐326 and increase the expression of cortactin through c‐Src signaling, leading to enhanced cervical cancer invasiveness (Cheng et al., 2018).

*VEGF* binds to the receptor tyrosine kinase (VEGFR) by transphosphatase, and then activates VEGFR. *VEGFR‐2* and *VEGFR‐3* are belongs to the tyrosine kinase receptor family (Masabumi, 2006). *VEGFR‐3* is activated by two proteins (*VEGF‐C* and *VEGF‐D*), and plays an essential role in the morphogenesis of the lymphatic vessel network during embryonic development, being involved in formation of new lymphatic vessels in the life. Binding of *VEGF‐C* to *VEGFR‐3* is responsible for mostly biological effects of *VEGFR‐3* (Olsson, Dimberg, Kreuger, & Claesson‐Welsh, 2006). Some studies have reported that *VEGFR‐2* may also interfere with lymphangiogenesis by binding *VEGF‐C* and *VEGF‐D*, which was crucial for the normal process of vasculogenesis during embryonic development (Ferrara, 2004; Vokes & Krieg, 2015). *VEGFR‐2* and *VEGFR‐3* are involved in normal and pathological angiogenesis through different mechanisms, such as: activation of MAPK extracellular signal‐regulated kinases (ERK1/2) through the PKC and Ras pathways (Shibuya, 2011; important pathways in cell proliferation), as well as the PI3K Akt/PKB pathway (involved especially in survival of lymphatic endothelial cells; Olsson et al., 2006). Moreover, it activates certain integrins, which disrupt cell to cell cohesion and initiate cellular migration (Takahashi & Shibuya, 2005).

In this study, we explored the relationship of *VEGF‐C*, *VEGFR‐2,* and *VEGFR‐3* polymorphisms with cervical cancer risk. There were significant associations between their polymorphisms and the risk of various diseases (hepatocellular carcinoma, oral cancer, gastric cancer, lymphedema, etc.; Chien et al., 2013; Debrah et al., 2017; Hsieh et al., 2014; Li, Yu, et al., 2018), except cervical cancer. We found that the rs4604006 of *VEGF‐C* gene was associated with an increased risk of cervical cancer, while rs12646659 was significantly associated with a decreased risk of cervical cancer for the first time. Meanwhile, there are few reports focused on the polymorphisms of rs4604006 and rs12646659. Only one study aimed to assess the role of *VEGF* and *VEGFR* polymorphisms in clinical outcomes of HCC patients receiving sorafenib therapy, and it found that rs4604006 (*VEGF‐C*) T allele was significantly associated with patients’ progression‐free survival and overall survival (Scartozzi et al., 2014). In addition, rs4604006 and rs12646659 were located in the intron region of *VEGF‐C* gene and may be involved in mRNA processing, and hence regulate posttranscriptional modification, protein translation, or promoter/enhancer cluster activity (Rose, 2008; Zhang et al., 2018). Besides, we will expand the sample size to verify our results and conduct further functional studies so as to provide more evidence for the effect of *VEGF‐C* polymorphism on cervical cancer risk.

Inevitably, this study had some limitations that should be accounted for when interpreting the results. First of all, the inherent selection bias and information bias were inevitable problems, because all participants were recruited from the identical hospitals. Second, the number of cases in our study was limited and our study population was all Chinese Uygur population, which cannot preclude false‐negative results and extrapolated to other populations. Hence, larger prospective studies are necessary to fully elucidate the role of these polymorphisms in cervical cancer. Despite these limitations, our current findings provide scientific evidence of *VEGFR‐2*, *VEGF‐C,* and *VEGFR‐3* with the risk of cervical cancer in the future studies.

## 5 CONCLUSION

To sum up, our study firstly provided evidence that the variants of *VEGF‐C* gene had a significant effect on the risk of cervical cancer in Chinese Uygur population, especially individuals older than 43. These results may contribute to further elucidate the potential role of *VEGF‐C* in cervical cancer susceptibility among Chinese Uygur population.

## CONFLICT OF INTEREST

The authors declare that they have no competing interests.
